# The Association between Obesity and Colorectal Cancer

**DOI:** 10.1155/2012/768247

**Published:** 2012-12-10

**Authors:** Kevin Whitlock, Richdeep S. Gill, Daniel W. Birch, Shahzeer Karmali

**Affiliations:** ^1^Faculty of Medicine & Dentistry, University of Alberta, Edmonton, AB, Canada T6G 2R3; ^2^Department of Surgery, University of Alberta, Edmonton, AB, Canada T6G 2R3; ^3^Center for the Advancement of Minimally Invasive Surgery (CAMIS), Royal Alexandra Hospital, Edmonton, AB, Canada T5H 3V9

## Abstract

Obesity has become a major issue for healthcare providers as its prevalence continues to increase throughout the world. The literature suggests that increased body mass index (BMI) is associated with the development of certain cancers such as colorectal cancer (CRC). Consequently, CRC surgeons are now encountering an increasing number of obese patients which may influence the technical aspects and outcomes of surgical treatment. For instance, obese patients present with greater comorbidities preoperatively, which adds increasing complexity and risks to surgical management. Recent literature also suggests that obesity may increase operating time and conversion rates to open colorectal surgery. Postoperative outcomes may also be influenced by obesity; however, this currently remains controversial. There is evidence that survival rates after CRC surgery are not influenced by obesity. In summary, obesity presents challenges to CRC surgeons, and further research will be needed to show how this important characteristic influences the outcomes for CRC patients.

## 1. Introduction

Globally, the adult population's body mass index (BMI) has been increasing over the past 30 years [1]. The World Health Organization estimates that 1.5 billion people are overweight and 500 million are obese worldwide [2]. This equates to 20% of the population being considered overweight and 10% defined as clinically obese [3]. Concurrently, colorectal cancer (CRC) is the 3rd most prevalent cancer in the world with an estimated incidence of 0.02% [4]. An estimated 1.2 million individuals are diagnosed with CRC annually. CRC also contributed to 8.1% of all cancer-related deaths in 2008 [4]. In North America CRC is the 4th most commonly diagnosed cancer each year [5, 6] and second among cancer-related deaths in North America [5, 6]. In this paper, we explore the emerging evidence suggesting an association of obesity and CRC. In addition, we will review the recent evidence on the impact of obesity on outcomes following colorectal surgery for CRC. 

## 2. Obesity and Colorectal Cancer

The association between obesity and CRC has been a subject of interest as the prevalence of obesity continues to increase. Three large and international systematic reviews have assessed this association, which is summarized in Table 1. Overall, there is increasing evidence that an increased BMI increases the likelihood of developing CRC. Though these large meta-analyses include heterogeneous populations, they are important to discuss. 

Firstly, Polednak conducted a meta-analysis of 153,760 Americans [7]. They reported that the relative risk (RR) for developing CRC was 1.4 for obese men and 1.1 for obese women. Interestingly, this suggests that the association is weaker for women when compared to men. The mechanism leading to a gender difference remains unclear, but it does suggest that there is likely a multitude of factors that influence whether an obese individual will develop CRC. The importance of understanding the association between CRC and obesity is related to the increasing prevalence of obesity, which suggests that a concurrent increase in prevalence of obesity-related cancers may occur. Since CRC is thought to be an obesity-related cancer, it would be reasonable to predict an increase in CRC incidence.

Secondly, Harriss et al. performed a meta-analysis to determine the relationship between obesity and CRC [8]. Their analysis included 28 studies with an international population of 67,361 CRC cases. They reported a greater association between obesity and colon cancer when compared to rectal cancer. As well, obese men had a relative risk of 1.24 for developing colon cancer, compared to obese women with a relative risk of 1.09. The author's analysis included confounding factors, such as physical activity, smoking, alcohol usage, and family history. Of these factors, the only statistically significant difference in risk ratio was seen in smoking women. These findings suggest that smoking may be a confounding variable in these association studies. 

The presumptive link between obesity and CRC remains challenging, as other confounding factors may influence the development of CRC. Fat distribution, gender, family history, substance use, and other comorbidities may influence CRC development in obese individuals. The current studies are relatively heterogeneous, which makes claims about the impact of obesity on incidence of CRC inconclusively; however, an association may exist. 

On the other hand, a direct link between obesity and CRC remains controversial. Coussens and Werb suggested that chronic inflammation secondary to obesity has an established association with cancer [9]. As well, increasing obesity can lead to insulin resistance [10] which has been reported to triple the risk of CRC mortality in a study of 62,285 individuals by Trevisan et al. [11]. Supportively, Colangelo et al. in a study of 35,582 individuals reported that insulin resistance increased the relative risk to 1.5 for mortality secondary to CRC [12]. Therefore, the influence of obesity on CRC may involve insulin resistance; although the current literature has shown associations, causality has not been proven. 

Interestingly, individuals who have undergone bariatric surgery were less likely to develop CRC [13]. Christou et al. reported that the relative risk was 0.32 for bariatric surgery patients developing CRC when compared to nonsurgery controls [13]. Further, in a prospective cohort study, Sjöström et al. included 4047 patients who either underwent bariatric surgery or received conventional weight loss strategies including medical and lifestyle interventions [14]. The purpose of their study was to compare cancer incidence in the surgical and conventional groups. Sjöström et al. found that the surgery group had a hazard ratio of 0.67 for overall cancer incidence in comparison to the control group. Unfortunately, this study did not distinguish between specific cancers. Hence, with limited literature, the impact of bariatric surgery on CRC incidence remains to be defined. 

## 3. Obesity-Related Comorbidities and Surgery

Obese individuals that develop CRC represent a unique patient population with a presumed higher prevalence of comorbid disease. A recent study by Merkow et al. included 3,202 patients that underwent a segmental colectomy for CRC at a total of 121 hospitals [18]. They reported that obese patients had a significantly higher prevalence of preoperative type-2 diabetes mellitus (T2DM) at 24.7% relative to nonobese patients at 9.2%. However, the prevalence of COPD, congestive heart failure, coronary artery disease, neurologic disease, and renal failure was not significantly different between the two groups. 

Interestingly, Healy et al. reported comparable preoperative conditions among obese patients [19]. Their prospective study included 414 patients who underwent elective surgery for CRC [19], and obese patients in their study had a significantly higher prevalence of preoperative T2DM and cardiovascular disease when compared to the nonobese patients. As well, Kamoun et al. recently reported a prospective review of 180 patients who underwent elective laparoscopic colorectal surgery at their institution [20] and reported that obese patients had significantly higher prevalence of preoperative cardiopulmonary comorbidities. Therefore, obese CRC patients appear to have increased prevalence of T2DM and cardiopulmonary disease. The influence of these comorbidities on surgical outcomes was assessed by Panis et al. [21]. These authors reported a multivariate analysis of 84,524 French CRC surgical patients [21]. Their study used prospective data from the National Health Services database and included analysis of medical comorbidities with respect to 30-day mortality. They classified comorbidities based on the World Health Organization International Classification of Diseases. Panis et al. reported that vascular comorbidities were associated with an increased 30-day postoperative mortality for CRC patients (odds ratio (OR) 2.66). Additionally, respiratory and neurologic comorbidities were associated with increased 30-day postoperative mortality (OR 3.13 and 1.78, resp.). Interestingly, T2DM did not significantly influence 30-day postoperative mortality. 

A Korean study by Noh et al. assessed survival and T2DM [22]. They looked at 657 CRC patients and reported a T2DM prevalence of 10%. As well, their diabetic patients had a significantly higher BMI relative to their nondiabetic patients. This is in agreement with the prior-mentioned studies that determined that obese patients had a higher preoperative prevalence of T2DM. After CRC surgery, Noh et al. found no significant difference in the recurrence-free survival rate or overall five-year survival rate when comparing diabetic to nondiabetic patients. As well, the mean survival in years after CRC surgery was statistically similar for the two groups (9.6 years for T2DM and 10.6 years for non-T2DM). 

There remains disagreement in the literature with respect to T2DM and CRC outcomes. Chen et al. performed a prospective analysis of 945 Chinese CRC patients, of which only 2.8% had T2DM [27]. They determined that T2DM was associated with a significantly lower disease-free survival rate (34.2%) when compared to non-T2DM patients (55.1%). Chen et al. concluded that T2DM increases the risk of CRC recurrence after surgical resection. However, Anand et al. reported conflicting results for T2DM and CRC [28]. They included 218,534 American CRC surgical patients from the Nationwide Inpatient Sample and observed a T2DM prevalence of 15%. Anand et al. then reported that T2DM patients actually had a 23% lower inhospital mortality rate and a significantly lower postoperative complication rate (adjusted OR 0.82) when compared to nondiabetics. Therefore, currently the majority of studies support the contention that T2DM does not negatively impact CRC surgical outcomes.

## 4. Obesity and Colorectal Surgery

Obese patients are increasingly presenting for surgical treatment and may impact intraoperative outcomes for CRC. The influence of obesity on operating times for colorectal procedures is controversial. In a retrospective study by Tsujinaka et al. of 133 patients who underwent elective laparoscopic sigmoid colectomy for sigmoid colon cancer [29], they reported that the mean operating time was significantly longer for obese patients (235 min) when compared to nonobese patients (207.5 min). Balentine et al. found similar increases in operating times for laparoscopic approaches to obese rectal cancer patients [30]. However, a retrospective study by Leroy et al., which looked at 123 patients undergoing laparoscopic left colonic resections, reported that obese and nonobese patients had similar operating times [31]. Interestingly, the retrospective review of 737 patients by Benoist et al. suggested that the type of surgery influenced the operating time [32]. For instance, obese patients undergoing a left colectomy had similar operating times to nonobese patients. However, obese patients undergoing a rectal resection had significantly longer operating times (375 min) in comparison to nonobese patients (310 min). 

In terms of surgical approach, a systematic review by Makino et al. [33] included 33 studies that reported higher rates of conversion from laparoscopic to open surgery for obese patients with CRC. The most common reasons for conversion were difficulties with dissection and exposure. A retrospective study by Park et al. [34], which included 984 patients who underwent CRC surgery, reported that obese patients had significantly more conversions to open surgery (14.8% versus 2.6%). Their discussion agreed with the conclusions of Makino et al. as they specifically described problems obtaining good exposure and the technical demands of operating on an obese patient.

## 5. Obesity and Colorectal Surgery Outcomes

Obesity may impact survival outcomes for CRC patients. Calle et al. examined the relationship of BMI and cancer-related deaths in a prospective cohort study of 900,053 American adults [35]. They observed a significant positive trend of increasing mortality from colorectal cancer with increasing BMI for both men and women. For instance, men with a BMI of 30.0–34.9 kg/m^2^ had a 1.47 relative risk of death from colorectal cancer compared to normal weight individuals. As well, men with a BMI ≥ 35.0 kg/m^2^ had a relative risk of death from colorectal cancer of 1.84 compared to normal weight individuals. 

However, a recent report on the trends associated with CRC in the United States conflicts with the outcomes reported by Eheman et al. [36]. Eheman et al. assessed mortality data from the Center for Disease Control (CDC) over a period of 9 years [36]. They reported that there has been a 3.0% decrease for men and a 2.9% decrease for women in CRC deaths between 1999 and 2008. However, the report by Eheman et al. was not able to exclude other variables that could impact mortality rates. Therefore, it is still possible that obesity contributes to an increased mortality rate for CRC patients, with overall mortality rates steadily decreasing from CRC. 

Merkow et al. also assessed postoperative complications following surgery in CRC patient's [18]. They reported that patients with class I obesity (30.0–34.9 kg/m^2^) had similar short-term complication rates relative to the nonobese patients. However, patients with class II/III obesity (BMI > 35.0 kg/m^2^) had significantly greater incidence of surgical site infections, pulmonary embolism, and wound dehiscence. Additionally, the prospective study by Akiyoshi et al. reported that class II obese CRC surgical patients had significantly more anastomotic leaks and wound infections in comparison to nonobese CRC patients [37]. However, the class I obese CRC patients in their study had similar surgical outcomes when compared to the nonobese patients. 

Contrastingly, a prospective study by Healy et al. did not observe a significant difference in wound complications, anastomotic leaks, or pulmonary embolism between obese and nonobese groups [19]. However, these authors did report that obese patients had a higher incidence of pelvic abscesses (5.3% versus 1.5%). Interestingly, a retrospective review of 69 patients by Sakamoto et al. [38] of patients that underwent laparoscopic colectomy for CRC reported that obese patients had similar postoperative complication rates as the nonobese patients. However, this study may have been underpowered to demonstrate a difference if one did indeed exist.

In contrast, Sakamoto et al. reported that significantly more nonobese patients required reoperations (2% versus 0%). Therefore, the effect that obesity has on postoperative complications remains unclear. This may be related to the fact that the literature does not consistently report on postoperative complications, making generalizations difficult. In addition, it is likely that other patient factors influence the study of obesity and postoperative outcomes. 

Interestingly, a recent review by Makino et al. reported that the mortality rate due to surgical complications was similar between obese and nonobese CRC patients [33]. Similarly, a retrospective review by Singh et al. [25], which included 234 patients that underwent laparoscopic surgery for CRC, reported that 30-day mortality rates were similar between the obese (8%) and nonobese patients (6%). Leroy et al. also reported similar mortality rates for obese and nonobese patients [31], in a retrospective review that included 123 patients who underwent laparoscopic left colonic resections. In contrast, a retrospective review by Benoist et al. reported that obesity increased the mortality rate from 0.5% to 5% [32]. However, this study was not exclusive to colorectal cancer patients. 

Based on the current evidence in the literature, obese patients likely have similar short-term mortality rates after surgery when compared to nonobese patients. It is also feasible that with low mortality rates following CRC, a statistical difference is difficult to reach with small patient populations in both retrospective and prospective studies. Also, with inconsistent reporting of postoperative outcomes and inclusion of obese patients ranging from Class I to Class III obesity, the link between obesity and postoperative course/complications is poorly understood.

## 6. Obesity and Oncologic Outcomes in ****Colorectal Cancer

Oncologic outcomes following surgery for CRC are the most crucial.  Table 2 summarizes the studies that have investigated these outcomes in obese CRC patients. In a prospective study by Healy et al., the presence of residual disease (based on pathology specimens) was similar in obese and nonobese patients [19]. In addition, these authors reported that five-year survival was similar between obese and nonobese patients (60%), and their five-year survival rate is consistent with the rate reported in the literature [39]. However, it should be noted that Healy et al. reported that only 4% of their patients had a BMI > 35 kg/m^2^ [19], with most of their obese patients having class I obesity. Sinicrope et al. reported a randomized trial of 4,381 colorectal cancer patients receiving various courses of adjuvant chemotherapy [23]. Sinicrope et al. found that obese patients had significantly worse-disease-free survival (hazard ratio 1.13) and overall survival (hazard ration 1.13) when compared to normal weight patients, after 8 years of followup. When they took gender into account, only class I obese women had a significantly worse overall survival (HR 1.24) when compared to nonobese women. On the other hand, only class II/III obese men had a significantly different survival rate (HR 1.35) compared to nonobese men. However, it should be noted that obese patients in their study had a significantly higher stage of cancer in comparison to the non-obese patients. 

There is a paucity of studies that have investigated the impact of obesity on oncologic outcomes. Unfortunately, the studies looking at survival rates have conflicting results. However, it appears that gender may also influence the survival rate of obese CRC patients. This variable was not analyzed by Healy et al., therefore it is difficult to know whether their results were truly different from Sinicrope et al.. The other controversial issue is the mechanism by which obesity would influence survival rates. Is obesity a surrogate marker for insulin resistance, altered hormone production, diet, exercise, or another factors? There remains limited evidence to provide patients with information on how their BMI influences their survival from CRC. Future studies must take into account both the different classes of obesity and gender as both these variables have been suggested to impact CRC survival rates.

## 7. Conclusion

The prevalence of obesity continues to increase worldwide, and the presence of CRC is encountered more often in these patients. With CRC being the 3rd most common cancer worldwide, an association with obesity has been suggested by a number of studies. There are still a number of controversies with respect to obesity and CRC surgery. There are limited data to suggest that obesity influences intraoperative outcomes. It is likely that technical challenges exist when central distribution of body weight, increased visceral obesity, and male gender are demographics features; however, it remains unclear if obesity clearly impacts morbidity and mortality. Additionally, there is a need to have more consistency among the types of postoperative outcomes reported in CRC surgery studies. A focus on major complications may allow for stronger conclusions about the influence of obesity on CRC surgical outcomes. Furthermore, oncologic outcomes are paramount in any study of surgical intervention for CRC. Currently only a select few studies have addressed this issue. Further research is needed to understand the effects of the changing demographics of our surgical CRC population.

## Figures and Tables

**Table 1:** Obesity and risk of developing colon cancer.

Study	Study type	Population	*N*	CRC risk obese versus nonobese	Conclusion
Dai et al. 2007 [15]	Meta-analysis	International	6,458	RR 1.37 for males	Obese men have increased risk of CRC

Polednak 2008 [7]	Meta-analysis	American	153,760	RR 1.4 for males RR 1.1 for females	Obese men have greater risk of CRC

Harriss et al. 2009 [8]	Meta-analysis	International	67,361	RR 1.24 for males RR 1.09 for females	Higher BMI increases risk of colon cancer. Men have greater risk than women.

Pischon et al. 2006 [16]	Prospective cohort	European	368,277	RR 1.55 for males RR 1.06 for females	Obese men have greater risk of CRC

Rapp et al. 2005 [17]	Prospective cohort	Austrian	145,000	HR 1.56 for males and colon cancer	Obese men have greater risk of CRC. Obese womens have greater risk of colon cancer relative to rectal cancer.
				HR 1.11 for females and colon cancer	
				HR 1.66 for males and rectal cancer	
				HR 0.66 for females and rectal cancer	

RR: relative risk.

HR: hazard ratio.

**Table 2:** Obesity and oncologic outcomes after CRC surgery.

Study	Study type	Population	*N*	Obese versus nonobese CRC patients	Conclusion
Healy et al. 2010 [19]	Retrospective cohort	British	414	No difference, 5-year survival 60% both groups	Class I obese CRC patients have similar survival rates compared to nonobese CRC patients

Sinicrope et al. 2010 [23]	Randomized controlled Trial	American	4,381	Survival difference, Class I obese women HR 1.24 versus nonobese, women	Overall CRC survival is influenced by BMI and Gender.
				Survival difference, Class II/III obese men HR 1.35 versus nonobese men	

Ballian et al. 2010 [24]	Retrospective cohort	American	254	Similar disease-free survival rates at 2 years postop (85% for obese and 76% for nonobese)	Obese rectal cancer patients have similar disease-free survival rates compared to nonobese rectal cancer patients

Singh et al. 2011 [25]	Retrospective cohort	British	234	Similar disease recurrence rates (8% for both obese and nonobese patients)	Obese CRC patients have similar disease recurrence rates compared to nonobese CRC patients

Yamamoto et al. 2012 [26]	Retrospective cohort	Japanese	273	Similar recurrence-free survival and overall survival after 72 months	Obese CRC patients have similar survival rates compared to nonobese CRC patients

## References

[1] Finucane MM, Stevens GA, Cowan MJ (2011). National, regional, and global trends in body-mass index since 1980: systematic analysis of health examination surveys and epidemiological studies with 960 country-years and 9*·*1 million participants. *The Lancet*.

[2] WHO Obesity and Overweight.

[3] Kelly T, Yang W, Chen CS, Reynolds K, He J (2008). Global burden of obesity in 2005 and projections to 2030. *International Journal of Obesity*.

[4] International Agency for Research on Cancer Globocan 2008 Fact Stats.

[5] Canadian Cancer Society Canadian Cancer Statistics 2011.

[6] American Cancer Society Cancer Facts & Figures 2011.

[7] Polednak AP (2008). Estimating the number of U.S. incident cancers attributable to obesity and the impact on temporal trends in incidence rates for obesity-related cancers. *Cancer Detection and Prevention*.

[8] Harriss DJ, Atkinson G, George K (2009). Lifestyle factors and colorectal cancer risk (1): systematic review and meta-analysis of associations with body mass index. *Colorectal Disease*.

[9] Coussens LM, Werb Z (2002). Inflammation and cancer. *Nature*.

[10] Ashrafian H, Ahmed K, Rowland SP (2011). Metabolic surgery and cancer. *Cancer*.

[11] Trevisan M, Liu J, Muti P, Misciagna G, Menotti A, Fucci F (2001). Markers of insulin resistance and colorectal cancer mortality. *Cancer Epidemiology Biomarkers and Prevention*.

[12] Colangelo LA, Gapstur SM, Gann PH, Dyer AR, Liu K (2002). Colorectal cancer mortality and factors related to the insulin resistance syndrome. *Cancer Epidemiology Biomarkers and Prevention*.

[13] Christou NV, Lieberman M, Sampalis F, Sampalis JS (2008). Bariatric surgery reduces cancer risk in morbidly obese patients. *Surgery for Obesity and Related Diseases*.

[14] Sjöström L, Gummesson A, Sjöström CD (2009). Effects of bariatric surgery on cancer incidence in obese patients in Sweden (Swedish Obese Subjects Study): a prospective, controlled intervention trial. *The Lancet Oncology*.

[15] Dai Z, Xu YC, Niu L (2007). Obesity and colorectal cancer risk: a meta-analysis of cohort studies. *World Journal of Gastroenterology*.

[16] Pischon T, Lahmann PH, Boeing H (2006). Body size and risk of colon and rectal cancer in the European Prospective Investigation into Cancer and Nutrition (EPIC). *Journal of the National Cancer Institute*.

[17] Rapp K, Schroeder J, Klenk J (2005). Obesity and incidence of cancer: a large cohort study of over 145 000 adults in Austria. *British Journal of Cancer*.

[18] Merkow RP, Bilimoria KY, McCarter MD, Bentrem DJ (2009). Effect of body mass index on short-term outcomes after colectomy for cancer. *Journal of the American College of Surgeons*.

[19] Healy LA, Ryan AM, Sutton E (2010). Impact of obesity on surgical and oncological outcomes in the management of colorectal cancer. *International Journal of Colorectal Disease*.

[20] Kamoun S, Alves A, Bretagnol F, Lefevre JH, Valleur P, Panis Y (2009). Outcomes of laparoscopic colorectal surgery in obese and nonobese patients: a case-matched study of 180 patients. *American Journal of Surgery*.

[21] Panis Y, Maggiori L, Caranhac G (2011). Mortality after colorectal cancer surgery: a French survey of more than 84,000 patients. *Annals of Surgery*.

[22] Noh GY, Hwang DY, Choi YH, Lee YY (2010). Effect of diabetes mellitus on outcomes of colorectal cancer. *Journal of the Korean Society of Coloproctology*.

[23] Sinicrope FA, Foster NR, Sargent DJ, O’Connell MJ, Rankin C (2010). Obesity is an independent prognostic variable in colon cancer survivors. *Clinical Cancer Research*.

[24] Ballian N, Yamane B, Leverson G (2010). Body mass index does not affect postoperative morbidity and oncologic outcomes of total mesorectal excision for rectal adenocarcinoma. *Annals of Surgical Oncology*.

[25] Singh A, Muthukumarasamy G, Pawa N, Riaz AA, Hendricks JB, Motson RW (2011). Laparoscopic colorectal cancer surgery in obese patients. *Colorectal Disease*.

[26] Yamamoto N, Fujii S, Sato T Impact of body mass index and visceral adiposity on outcomes in colorectal cancer.

[27] Chen CQ, Fang LK, Cai SR (2010). Effects of diabetes mellitus on prognosis of the patients with colorectal cancer undergoing resection: a cohort study with 945 patients. *Chinese Medical Journal*.

[28] Anand N, Chong CA, Chong RY, Nguyen GC (2010). Impact of diabetes on postoperative outcomes following colon cancer surgery. *Journal of General Internal Medicine*.

[29] Tsujinaka S, Konishi F, Kawamura YJ (2008). Visceral obesity predicts surgical outcomes after laparoscopic colectomy for sigmoid colon cancer. *Diseases of the Colon and Rectum*.

[30] Balentine CJ, Wilks J, Robinson C (2010). Obesity increases wound complications in rectal cancer surgery. *Journal of Surgical Research*.

[31] Leroy J, Ananian P, Rubino F, Claudon B, Mutter D, Marescaux J (2005). The impact of obesity on technical feasibility and postoperative outcomes of laparoscopic left colectomy. *Annals of Surgery*.

[32] Benoist S, Panis Y, Alves A, Valleur P (2000). Impact of obesity on surgical outcomes after colorectal resection. *American Journal of Surgery*.

[33] Makino T, Shukla PJ, Rubino F (2012). The impact of obesity on perioperative outcomes after laparoscopic colorectal resection. *Annals of Surgery*.

[34] Park JW, Lim SW, Choi HS, Jeong SY, Oh JH, Lim SB (2010). The impact of obesity on outcomes of laparoscopic surgery for colorectal cancer in Asians. *Surgical Endoscopy and Other Interventional Techniques*.

[35] Calle EE, Rodriguez C, Walker-Thurmond K, Thun MJ (2003). Overweight, obesity, and mortality from cancer in a prospectively studied cohort of U.S. Adults. *New England Journal of Medicine*.

[36] Eheman C, Henley SJ, Ballard-Barbash R (2012). Annual Report to the Nation on the status of cancer, 1975–2008, featuring cancers associated with excess weight and lack of sufficient physical activity. *Cancer*.

[37] Akiyoshi T, Ueno M, Fukunaga Y (2011). Effect of body mass index on short-term outcomes of patients undergoing laparoscopic resection for colorectal cancer: a single institution experience in Japan. *Surgical Laparoscopy Endoscopy & Percutaneous Techniques*.

[38] Sakamoto K, Niwa S, Tanaka M, Goto M, Sengoku H, Tomiki Y (2007). Influence of obesity on the short-term outcome of laparoscopic colectomy for colorectal cancer. *Journal of Minimal Access Surgery*.

[39] Andreoni B, Chiappa A, Bertani E (2007). Surgical outcomes for colon and rectal cancer over a decade: results from a consecutive monocentric experience in 902 unselected patients. *World Journal of Surgical Oncology*.

